# Applications of protein ubiquitylation and deubiquitylation in drug discovery

**DOI:** 10.1016/j.jbc.2024.107264

**Published:** 2024-04-04

**Authors:** Yilin Chen, Haoan Xue, Jianping Jin

**Affiliations:** 1 Life Sciences Institute, Zhejiang University, Hangzhou, China; 2 Center for Life Sciences, Shaoxing Institute, Zhejiang University, Shaoxing, China; 3 Cancer Center, Zhejiang University, Hangzhou, China

**Keywords:** PROTAC, molecular glue, DUBTAC, ubiquitin, ubiquitylation, deubiquitylation

## Abstract

The ubiquitin (Ub)–proteasome system (UPS) is the major machinery mediating specific protein turnover in eukaryotic cells. By ubiquitylating unwanted, damaged, or harmful proteins and driving their degradation, UPS is involved in many important cellular processes. Several new UPS-based technologies, including molecular glue degraders and PROTACs (proteolysis-targeting chimeras) to promote protein degradation, and DUBTACs (deubiquitinase-targeting chimeras) to increase protein stability, have been developed. By specifically inducing the interactions between different Ub ligases and targeted proteins that are not otherwise related, molecular glue degraders and PROTACs degrade targeted proteins *via* the UPS; in contrast, by inducing the proximity of targeted proteins to deubiquitinases, DUBTACs are created to clear degradable poly-Ub chains to stabilize targeted proteins. In this review, we summarize the recent research progress in molecular glue degraders, PROTACs, and DUBTACs and their applications. We discuss immunomodulatory drugs, sulfonamides, cyclin-dependent kinase–targeting molecular glue degraders, and new development of PROTACs. We also introduce the principle of DUBTAC and its applications. Finally, we propose a few future directions of these three technologies related to targeted protein homeostasis.

Proteins play important roles in transducing cellular signals and carrying out specific tasks in various biological processes. When they accomplish their duties, proteins must be inactivated or even degraded to avoid potential obstacle to life activities. For example, accumulation of misfolded proteins leads to degenerative diseases (1) and oncogenic proteins lead to tumorigenesis when overexpressed (2). That is why disease-prone proteins have been considered as therapeutic targets.

Small-molecule drugs are among the most effective weapons employed toward disease therapeutics, but not all disease-relevant proteins can be easily targeted, especially those without active regions using traditional drugs like small-molecule inhibitors. More importantly, mutations can often occur in targeted proteins to escape from inhibition by small-molecule drugs, especially in cancer cells. To solve these problems, new therapeutic approaches have been recently developed to induce degradation of disease-prone proteins, called targeted protein degradation (TPD) or stabilize disease-inhibitory proteins using the innate ubiquitylation system (3, 4, 5, 6, 7).

In this article, we will discuss three ubiquitin (Ub)–proteasome system (UPS)–based new technologies, including molecular glue degraders, PROTACs (proteolysis-targeting chimeras) (3), and DUBTACs (deubiquitinase-targeting chimeras). Since there are many reviews discussing PROTACs, we will focus more on molecule glue degraders and DUBTACs, a newly created strategy enhancing protein stability (7).

## Ub and ubiquitylation

**Figure 1:**
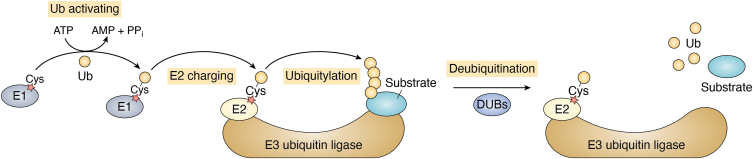
**Ubiquitylation and deubiquitylation.** Ubiquitylation is an enzymatic process depending on an E1–E2–E3 cascade, by which ubiquitin can be covalently attached to protein substrates. Ubiquitylation can be reversed by deubiquitylation, which is mediated by DUBs. DUB, deubiquitinase.

There are two major protein degradation systems in eukaryotic cells: the UPS and the autophagy lysosomal pathway (8, 9, 10, 11). Ub is a small but powerful protein that can be covalently attached to protein substrates through an isopeptide bond between the last C-terminal glycine residue of Ub and often a lysine residue on substrates (9, 10). Protein ubiquitylation (also called ubiquitination) depends on an enzymatic cascade involving an E1 (Ub-activating enzyme), an E2 (Ub-conjugating enzyme), and an E3 (Ub ligase) (9, 10, 12, 13, 14) (Fig. 1). Interestingly, Ub, as a protein itself, can be ubiquitylated as well. There are seven lysine residues on the surface of Ub, all of which can be involved in the formation of Ub chains (12, 13, 14). Besides, linear Ub chain can be formed *via* a peptide bond between the C-terminal glycine residue and the N-terminal methionine residue of two Ub molecules, respectively (12, 13, 14). Recent studies indicated that Ub can be conjugated to serine residues or threonine residues of protein substrates and Ub to form atypical poly-Ub chains (13, 14). Furthermore, heterotypic Ub chains, including branched and mixed chains, bring more complexity to Ub codes (12, 13, 14). Different Ub modifications form various Ub codes leading to diverse destinies of ubiquitylated proteins.

In general, protein ubiquitylation leads to two main consequences: one is proteolysis, and the other is involved in various nondegradative events, including protein localization changes, endocytosis, DNA damage repair, immune responses, and so on (9, 10). Typical degradation signals like K48-, K11-, as well as K48/K11-branched Ub chains are often related to proteolysis via the 26S proteasome (12). Nondegradative functions of Ub can be observed in different kinds of signaling pathways. For instance, M1- and K63-Ub chains take part in the NF-κB signaling pathway (12); K6- and K63-poly-Ub chains are observed during the Parkin-mediated mitophagy (15).

## Ub ligases largely determine the specificity of ubiquitylation

**Figure 2:**
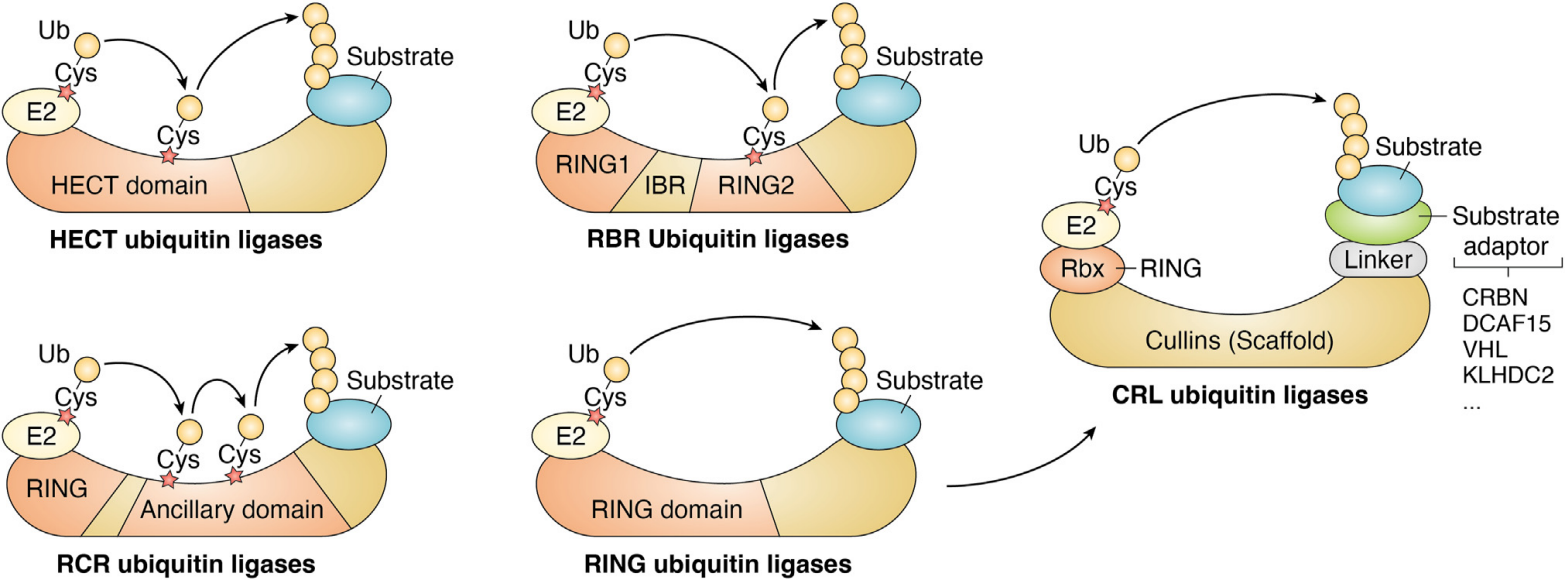
**Ubiquitin (Ub) ligase classification and the Cullin–RING Ub ligases (CRLs).** The specificity of protein ubiquitylation is mainly determined by Ub ligases, which can be divided into four types based on their typical domains and whether they possess enzymatic activities. Among them, there are three families harboring at least one active cysteine residue with enzymatic activities, including HECT, RBR, and RCR Ub ligases. Ub is conjugated to protein substrates by these Ub ligases directly. In contrast, RING Ub ligases, without enzymatic activities, function as scaffolds to shorten the distance between an active E2 enzyme and substrate to trigger ubiquitylation. In this case, Ub is transferred from E2 to its substrates directly. The biggest subfamily of RING Ub ligases is the CRLs, which contain multiple subunits, including RBX1 or RBX2 as a RING finger protein, one of the Cullins as a scaffold, a linker protein or a domain, and a substrate adaptor. Small-molecule ligands of CRLs are the most popular ones employed in PROTAC design. HECT, homologous to E6AP C terminus; PROTAC, proteolysis-targeting chimera; RBR, RING-between-RING; RCR, RING-Cys-relay.

**Figure 3:**
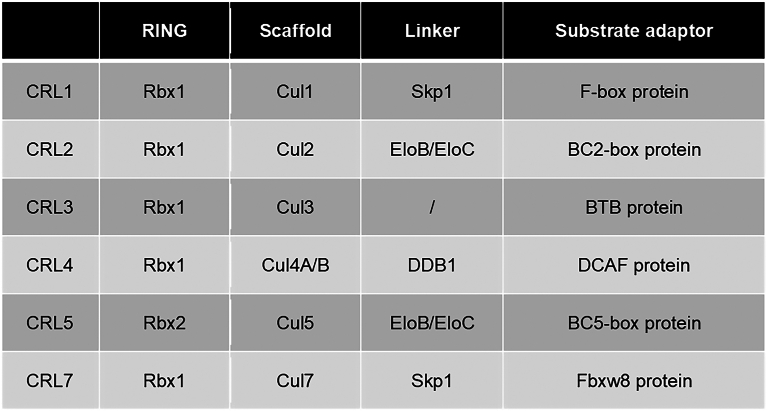
**Subunits of Cullin–RING ubiquitin ligases (CRLs).** There are two RING finger and seven cullin proteins involved in the organization of CRLs. In addition, linker proteins or a BTB (broad-complex, Tramtrack, and Bric-abrac) domain are required for cullins to connect to their corresponding substrate adaptors.

Ubiquitylation is a well-coordinated and specific process (9, 10), and the specificity is mainly determined by Ub ligases, which are encoded by over 600 E3 genes in the human genome. There are four families of Ub ligases: HECT (homologous to E6-AP carboxyl terminus), RING finger, RBR (RING-Between-RING), and RCR (RING-Cys-relay) domain Ub ligases (9, 10, 16, 17) (Fig. 2). The biggest subfamily of the RING finger Ub ligases is the Cullin–RING Ub ligase (CRL) (18, 19) (Fig. 2), which is composed of multiple subunits: proteins harboring RING domain for binding E2∼Ub (Rbx1 or Rbx2), scaffold protein (Cul1, Cul2, Cul3, Cul4A/B, Cul5, and Cul7), linker proteins (like DDB1 [damage-specific DNA-binding protein 1] and Skp1), and substrate adaptors, which recognize substrates specifically (Fig. 3). Nowadays, ligands of the CRLs are the most widely used small molecules in designing protein degradation drugs.

## Molecular glue degraders

**Figure 4:**
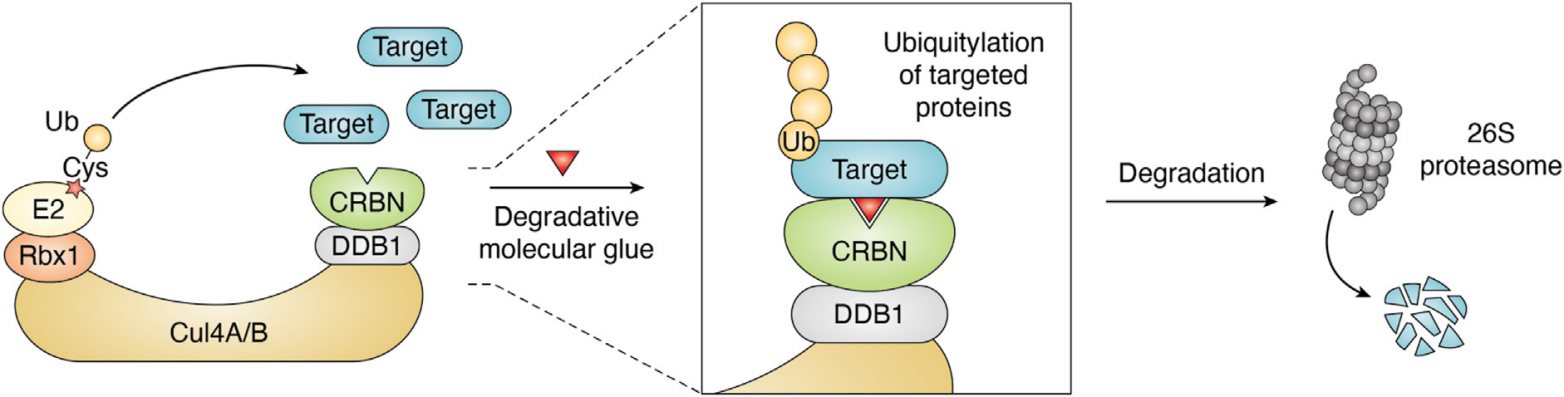
**CRL4**^**CRBN**^**-based molecular glue degrader.** Molecular glues are designed to achieve protein interactions. When used in protein degradation, molecular glues function as degraders to promote the interaction between ubiquitin ligases and targeted proteins and subsequent ubiquitylation and degradation of targeted proteins. A typical example is the CRL^CRBN^ ubiquitin ligase whose ligands (IMiDs) have been employed as either molecular glue degraders or ligands in PROTAC design. CRBN, cereblon; CRL, Cullin–RING ubiquitin ligase; IMiD, immunomodulatory drug; PROTAC, proteolysis-targeting chimera.

There are over 300,000 protein–protein interactions detected in human cells (20). Proximity influences many aspects of cell biology through mediating these protein–protein interactions. The concept of chemical inducers of proximity (CIPs) was initially aimed at the activation of signaling pathway by enhancing protein–protein interactions (21). Molecular glues are one kind of monovalent small-molecule CIPs with an ability to trigger protein–protein interactions. By specifically enhancing interactions between Ub ligases and targeted proteins, molecular glues can also work as degraders, meaning that drugs can trigger targeted protein turnover so that the problems like drug-escaping mutations and the absence of catalytic region can be bypassed (Fig. 4).

The first molecular glue degrader ever revealed is the plant hormone auxin, which was shown to trigger proteolysis of Aux/IAA transcription repressors by the SCF^TIR1^ Ub ligase in plants (22). Two studies further concluded that TIR1 is an auxin receptor *via* direct binding with auxin (23, 24). Tan *et al.* (25) then solved the structure of the full-length TIR1–ASK1 protein complex, where ASK1 acts as the adaptor protein of TIR1. By analyzing the structure of the TIR1–ASK1 complex interacting with three different auxin compounds and an Aux/IAA substrate peptide, they revealed the mechanism by which auxin promotes ubiquitylation and then degradation of Aux/IAA transcription repressors *via* the activities of the SCF^TIR1^ Ub ligase. The binding of auxin onto TIR1 causes little allosteric changes. Instead, they observed a surface top pocket in the TIR1 leucine-rich repeat domain for recruitment of both auxin and the substrate. Auxin binds at the bottom of this pocket, extending the interacting interface for Aux/IAA substrates by providing a hydrophobic base so that the catalytic activity of SCF^TIR1^ toward Aux/IAA can be largely enhanced. Thus, auxin has been considered as the first natural molecular glue degrader ever discovered.

Since then, the concept of molecular glue degraders has come into scientists’ view, although identifying more molecular glue degraders is not easy. In other words, most molecular glue degraders were found unintentionally. However, finding one molecular glue degrader can usually indicate typical functions of its analogs, and structural analysis further helps accelerate the discovery of its derivatives as new molecular glues that degrade the same or even new targeted proteins. Up to now, at least three kinds of molecular glue degraders have been found for drug discovery: thalidomide and its analogs/derivatives, aryl sulfonamides, and cyclin-dependent kinase (CDK) inhibitors.

### Thalidomide and its analogs/derivatives

Thalidomide was initially developed as a sedative but abandoned because of its teratogenic effects during early pregnancy. Its derivatives, including lenalidomide and pomalidomide, are relatively safe immunomodulatory drugs (IMiDs) for patients with cancer if not applied to pregnant women. In fact, lenalidomide has been a top 10 drug by sales in the world during recent years. However, the functional mechanism of IMiDs remained less clear for a period (26, 27, 28). In 2010, Ito *et al.* (29) reported that the primary target of thalidomide is a protein called cereblon (CRBN), a substrate adaptor in the CRL4 Ub ligase complex. In the following years, several studies further showed that analogs/derivatives of thalidomide including lenalidomide and pomalidomide also perform immunomodulatory activities through direct binding with CRBN (30, 31, 32). Although the initial study observed inhibition of Ub ligase activity after IMiDs’ binding to CRBN, subsequent findings identified specific substrates ubiquitylated by these IMiD-bound CRL4^CRBN^ Ub ligases, and then degraded in the 26S proteasome (33, 34, 35, 36, 37, 38, 39, 40, 41). More importantly, two key findings uncovered the underlying mechanisms of the teratogenic effects when used in pregnant women (39, 41).

Despite the teratogenic effects during early pregnancy, the roles of thalidomide and its analogs/derivatives as IMiDs applied to nonpregnant patients have been constantly studied with the discovery of many specific substrates of IMiD-bound CRL4^CRBN^ Ub ligases.

Krönke *et al.* (33) conducted a stable isotope labeling by/with amino acids in cell culture (SILAC)–based quantitative mass spectrometry (MS) study in the lenalidomide-treated MM1S multiple myeloma cell line. They identified IKZF1 (Ikaros) and IKZF3 (Aiolos) ranking at the top list of protein ubiquitylation and degradation changes. Using a truncation strategy, the authors confirmed a degron sequence in IKZF3 composed of 59 amino acids in its β-hairpin zinc finger (ZnF) domain 2. Although belonging to the same protein family, IKZF2 and IKZF4 are resistant to lenalidomide-induced degradation. In fact, only one amino acid in IKZF2/4 was found to be different from the corresponding amino acid in the IKZF1/3 ZnF2 domain (Q147 in IKZF3 and Q146 in IKZF1 *versus* H141 in IKZF2 and H188 in IKZF4), indicating that glutamine is key for the specificity of lenalidomide. Consistent to this finding, IKZF3^Q147H^ lost the degradation properties regulated by lenalidomide, and IKZF3 mutant-expressing cells exhibited resistance to lenalidomide-mediated cell growth inhibition. Meanwhile, Lu *et al.* (34) screened an ORF-luciferase library to search changes in protein stability affected by lenalidomide in 293FT cells and identified IKZF3 as the target of lenalidomide. They also found that IKZF1, the paralog of IKZF3, but not IKZF2, IKZF4, IKZF5, or the B-cell transcription factor (TF) IRF4, was degraded by lenalidomide, indicating the specificity of lenalidomide as a molecular glue degrader. Gandhi *et al.* in another study collected human peripheral blood mononuclear cell lysates treated with or without CRBN-binding glutarimide-containing analog and then performed immunoprecipitation using the di-glycine–lysine antibody and MS analysis. After analyzing peptides specifically ubiquitylated under thalidomide analog treatment, they identified IKZF3 as a substrate. They also confirmed the proteasome-dependent degradation of IKZF1 and IKZF3 by both lenalidomide and pomalidomide. All these studies revealed both IKZF1 and IKZF3 are protein substrates of the IMiD-bound CRL4^CRBN^ Ub ligases.

IMiD-mediated substrates are not limited to IKZF1 and IKZF3. To find out the mechanism by which lenalidomide is an excellent therapeutic drug for myelodysplastic syndrome patients with deletion of chromosome 5q (del(5q)), Krönke *et al.* (36) utilized the SILAC-based quantitative MS method in the del(5q) myeloid cell line KG-1 and discovered casein kinase 1A1 (CK1α) as a new substrate of the lenalidomide-bound CRL4^CRBN^ Ub ligase. They compared degradation activities of thalidomide, lenalidomide, pomalidomide, as well as a new analog named CC-122. Although these analogs of thalidomide all exhibit abilities to trigger IKZF1 degradation, only lenalidomide possesses the degradative effect toward CK1α, suggesting that a small difference among different drugs is enough to alter substrate specificity, further demonstrating the substrate specificity of these IMiD degraders.

Further structural analysis of DDB1–CRBN–IMiDs not only displayed important binding details of the complex but also identified MEIS2 as an endogenous substrate of CRL4^CRBN^ (32). IMiDs bind in a shallow hydrophobic surface pocket of CRBN *via* a glutarimide ring, which is structurally shared among these drugs. Then the newly formed interface on CRBN–IMiD complex leads to a switch from MEIS2 to IKZF1/3 or CK1α as new substrates. Petzold *et al.* (37) specifically analyzed the crystal structure of DDB1–CRBN–lenalidomide–CK1α complex and demonstrated that a β-hairpin loop in CK1α is responsible for its binding to the newly formed interface on DDB1–CRBN–lenalidomide. This is consistent to the findings mentioned previously (32), that is, the β-hairpin ZnF degron sequence in IKZF1/3 is important for CRL4^CRBN^–lenalidomide-induced degradation and cellular phenotypes, suggesting the potential of identifying more substrates of CRL4^CRBN^–IMiD based on this degron motif.

Sievers *et al.* (38) screened the human C2H2 ZnF proteome and identified 11 ZnF degrons: six of them could drive the turnover of their respective full-length proteins. They also found that different thalidomide analogs promote proteolysis of distinctive groups of ZnF proteins. Furthermore, combined with computational analysis and biochemical assays, they predicted that at least 150 ZnF proteins could bind to DDB1–CRBN–IMiD complexes and their proteolysis could be achieved by chemical modifications of these IMiDs and derivatives.

More CRBN-based molecular glue degraders were discovered in the next several years. Matyskiela *et al*. (39) designed screening experiments to look for CRBN ligands and identified the chemical CC-885. CC-885 exhibited antitumor activities in patient-derived acute myelogenous leukemia tumor cells with subnanomolar potency toward patients’ samples. Like the other IMiDs, CC-885 possesses a glutarimide ring, which is responsible for the binding with CRBN. Anti-FLAG affinity purification and MS analysis of human embryonic kidney 293T cells, which express FLAG-HA-CRBN, screened out GSPT1 (eRF3a) as a binding protein only in the presence of CC-885. Further studies showcased that CC-885, but not the other IMiDs, is able to mediate the binding of GSPT1 to CRBN and regulates proteasomal degradation of GSPT1, although all these chemicals can enhance the interaction between CRBN and IKZF1. Consistently, the antitumor effect of CC-885 is dependent on its regulation of GSPT1 degradation. Interestingly, there are little structural and sequence similarities between GSPT1 and other substrates of CRBN–IMiDs. However, some of the surface mutations on CRBN that abolished the CC-885-dependent recruitment of GSPT1 to CRBN showed similar inhibitory effect on the interaction between IKZF1 and CRBN, suggesting a common substrate recruitment manner of CRBN. For example, side chains of N351, H357, and W400 residues of CRBN were shown to form hydrogen bonds with GSPT1, which are also important for CRBN’s binding to IKZF1. The ZnF domain of IKZF1 was demonstrated to be necessary for providing a surface turn when binding with CRBN-CC-885. This structure feature can also be found in GSPT1, and it positions the three backbone hydrogen bonds at the top of this turn, with a necessary glycine residue at the key position, which is the only similarity between the sequence of GSPT1 and IKZF1 in this region. Since this substrate recognition model is dependent on the protein backbone rather than side chains, the potential of substrate sequence tolerance might exist if the backbone conformation remains the same. This study also extends the key residues in IKZF1 involved in the drug-dependent CRBN binding. Apart from Q146, G151 is also important because the mutation of G151A abolished the CC-885-dependent binding with CRBN. An *et al.* (40) later identified ZFP91 as the ubiquitylation substrate of lenalidomide-bound CRL4^CRBN^ by utilizing a pulse-chase SILAC MS-based proteomics approach. Sequence analysis revealed the existence of ZnF motif in ZFP91, where a key glycine residue in ZnF4 can be found. These findings further confirmed that these substrates bind with CRL4^CRBN^–IMiDs through its β-hairpin loop, as observed in IKZF1/3 and CK1α.

### Aryl sulfonamides

Indisulam (E7070) was initially discovered as an antitumor drug since it disturbs the G1 phase of the cell cycle in many tumor cell lines as well as xenograft mouse models (41, 42, 43).

In 2017, Han *et al.* (44) identified a nuclear protein RBM39 (RNA-binding motif protein 39) whose mutations lead to cancer cell resistance to indisulam treatment using a forward genetic strategy. They found that indisulam binds to DCAF15, the substrate adaptor in the CRL4^DCAF15^ Ub ligase. This binding results in the recruitment of RBM39 to this complex and its subsequent ubiquitylation and degradation. The absence of RBM39 blocks pre-mRNA splicing, thus inhibiting tumor progression. Moreover, the authors showcased a consistent working model of two other aryl sulfonamides drugs including tasisulam and chloroquinoxaline sulfonamide, which harbor similar structures with indisulam. These aryl sulfonamide drugs/sulfonamides targeting mRNA splicing are called splicing inhibitor sulfonamides.

E7820, also named NSC719239, is another kind of sulfonamide with antigrowth effects (45). It also works as a molecular glue degrader (46). In this case, the degradation of U2AF-related splicing factor coactivator of activating protein-1 and estrogen receptors (CAPERα) triggered by E7820 as well as indisulam and chloroquinoxaline sulfonamide was observed, indicating that more substrates of CRL4^DCAF15^-aryl sulfonamides might exist. Indeed, one study by Kim *et al.* (47) reported that the TF HIF1β directly interacts with DCAF15. The treatment with indisulam and E7820 induced HIF1β degradation through CRL4^DCAF15^ and inhibited its transcription activity. Another study by Jia *et al.* (48) integrated the pSILAC method together with the combination of LysC–trypsin and LysN–LysArgiNase digestion approaches to identify substrates of indisulam, directing the finding of pre-mRNA splicing factor PRPF39 as the substrate of the indisulam-bound CRL4^DCAF15^ Ub ligase. This pSILAC method focused on protein turnover with higher sensitivity compared with total protein quantification-based MS. Using the combination of LysC–trypsin and LysN–LysArgiNase, some peptides that are vulnerable to LysC–trypsin digestion can be recognized. Further structural analysis is still needed for a deeper comprehension of these degradation events.

Du *et al.* solved the crystal structure of DDA1 (DDB1 and DET1 associated 1)–DDB1–DCAF15–E7820–RBM39 complex, in which E7820 positions in a surface pocket on DCAF15 to form a new interface that binds RBM39 through its α-helix in the RRM2 domain. This new interface differs from the structure model of CRL4^CRBN^–IMiDs in complex with their substrates *via* β-hairpin motif on these targeted proteins (49). Moreover, kinetic studies indicated a synergistic binding manner of the complex. No interaction was observed between the RRM1–RRM2 (R1R2) domain of RBM39 and E7820 or indisulam. DCAF15 weakly binds E7820 and indisulam or the R1R2 domain independently. However, the presence of E7820 or indisulam decreases *K*_*d*_ values from ∼4.6 μM to 0.16 μM and 0.12 μM, respectively. The R1R2 domain decreases the complex-forming EC_50_ values of these two drugs. This study also demonstrated that the substrates of the E3–molecular glue complex are not necessarily the direct binding targets of individual molecular glue drugs, which suggested the difficulty of designing molecular glues for specific substrates or identifying the substrates of specific molecular glue candidates.

Considering that RBM39 interacts with DCAF15–E7820 through its conserved α-helix, Faust *et al.* (50) proposed the possibility that other proteins harboring the conserved α-helices in the RRM domain can also be the targets of DCAF15–E7820. Using MS analysis, they identified RBM23 as another substrate of DCAF15–E7820. Bussiere *et al.* (51) showed similar structural pattern of DDA1–DDB1–DCAF15–indisulam–RBM39 (RRM2) complex and identified RBM23 as a new substrate based on the α-helix degron motif, further confirming the recognizing rules of this kind of molecular glue degraders.

Although structurally unrelated to aryl sulfonamides, dCeMM1 was identified as a DCAF15 molecule glue to degrade RBM39 (52). However, no information is currently available to see whether it could also be a molecule glue degrader of other RBM domain–containing proteins.

### CDK molecular glue degraders

CDKs belong to the family of kinases involving regulations of cell cycle and transcription (53, 54, 55, 56). They are often dysregulated in tumorigenesis and have been examined extensively as therapeutic targets of cancers (57, 58, 59, 60). While small-molecule inhibitors were approved or under clinical development, scientists only began creating molecular glue degraders of CDKs.

By analyzing the correlation between small-molecule resistance of cancer cell lines and mRNA levels of Ub ligase components, Słabicki *et al.* (57) identified CR8, a CDK inhibitor, as the candidate molecular glue degrader for cyclin K (also termed CCNK). The cytotoxicity of CR8 was shown in correlation with the mRNA level of the CUL4 linker protein DDB1. Interestingly, no DCAF proteins were identified as substrate adaptors in their screening results, suggesting a direct interaction between CR8 and the components of the RBX1–CUL4–DDB1 complex, later revealed to be DDB1. The weak interaction between CDK12–cyclin K and DDB1 was largely enhanced by CR8, in which the DCAF binding domain of DDB1 is sufficient for the interaction. CDK12 plays a role of the substrate adaptor in a CR8-dependent manner, which then recruits cyclin K, resulting in CRL4-dependent ubiquitylation and degradation of cyclin K. CR8 is a CDK pan-inhibitor with a broad inhibitory effect toward other members of the CDK family, such as CDK9, CDK13, and so on. Meanwhile, cyclin K is also able to bind CDK9 and CDK13 besides CDK12. However, CR8-induced recruitment onto DDB1 is restricted to CDK12 and CDK13, instead of CDK9. Primary sequence comparison suggested that the C-terminal extension of CDK12 and CDK13 represents the specificity of DDB1’s binding to CDK12 or CDK13. However, disturbance of this region showed little impact on the drug-induced complex formation, suggesting that the C-terminal extension assists binding, but is not essential for drug-triggered kinase recruitment. The authors observed that CR8 interacts with the ATP-binding pocket of CDK12 and binds the BPC domain of DDB1 *via* its hydrophobic phenylpyridine ring. This study first proposed that molecular glue degraders can also interact directly with DDB1 without the help of any substrate adaptors, providing new avenues for molecular glue degrader design.

Based on chemical screening in hyponeddylated cells with widely impaired CRL function coupled to a quantitative expression proteomics approach, Mayor-Ruiz *et al.* (52) found three molecule glue degraders for cyclin K, including dCEMM2, dCEMM3, and dCEMM4. These three degraders are also capable of destabilizing CDK12 and CDK13, although with lower efficacies. Again, these molecule glues appear to be independent of any substrate adaptors in the CRL4 Ub ligases but are strongly dependent on DDB1, Cul4B, a neddylation E2 enzyme UBE2M, and UBE2G1, a Ub E2 enzyme that synthesizes poly-Ub chains. Interestingly, Cul4B is selected by these molecule glues as a favored scaffold protein over Cul4A, suggesting the uniqueness of these compounds in the selection of cullin scaffolds.

Another study by Lv *et al.* (58) identified the chemical HQ461 as the molecular glue degrader of CDK12–cyclin K, resulting in the proteasome degradation of cyclin K *via* the RBX1–CUL4–DDB1 Ub ligase. Originating from a small molecule screen searching for NRF2 inhibitors, HQ461 exhibited cytotoxicity toward the A549 cell line with IC_50_ value of 1.3 μM. Further loss-of-function and gain-of-function screens revealed that the components of the DDB1–CUL4–RBX1 Ub ligase complex and CDK12 gene mutations are related to the cytotoxicity of HQ461. More importantly, HQ461 treatment leads to 50% reduction in the CDK12 protein level, but modest CDK12 downregulation by CRISPR–Cas9 knockout technology failed to phenocopy the cell death induced by HQ461. Instead, cyclin K degradation is much faster than CDK12 proteolysis caused by HQ461, which can be inhibited by the expression of HQ461-resistant mutant CDK12. The HQ461-resistant mutations (G731E and G731R) of CDK12 are located within the central kinase domain of CDK12; however, G731E mutant or G731R mutant does not interfere with HQ461’s binding to CDK12 and instead disables DDB1 recruitment, explaining why these HQ461-resistant mutants could not promote cyclin K ubiquitylation and degradation.

Dieter *et al.* (59) discovered that compound NCT02 could also trigger cyclin K ubiquitylation and degradation. They screened through a library with ∼80,000 noncharacterized small molecules and aimed to identify inhibitory compounds toward all kinds of colorectal cancer cell subtypes in 24 primary colorectal cancer tumor spheroid culture cells. After confirming the inhibitory specificity of candidate compounds against patient-derived cancer cells rather than normal primary fibroblasts, Dieter *et al.* identified NCT02. Apart from inducing apoptosis and DNA damage inhibitory effect, the authors found that the cyclin K–CDK12 complex is the target of NCT02 using a thermal proteome profiling. NCT02 was observed to occupy the ATP-binding pocket of CDK12, functioning as a molecular glue to enhance the interaction between DDB1 and CDK12, and then triggering cyclin K degradation *via* the CRL in a DDB1-dependent manner.

NCT02, HQ461, and dCeMM4 do share a common *N*-(5-methylthiazol-2-yl) acetamide component, which occupies the ATP-binding pocket of CDK12. Besides, there are no common structural features that could recruit DDB1 among these molecule glue degraders, although structural docking models suggested that NCT02 and CR8 share similar binding modes with the CDK12–cyclin K–DDB1 complex.

Molecular glue degraders are not typical inhibitors of Ub ligases. Instead, they alter substrate specificity of Ub ligases, that is to say, they usually block the interactions between Ub ligases and their endogenous cognate substrates, leading to the ubiquitylation and proteolysis of neosubstrates. In addition, molecular glue degraders could be small molecule ligands in the construction of PROTACs to promote ubiquitylation and turnover of even more neosubstrates.

## PROTAC degraders

PROTACs are small molecule–based heterobifunctional protein degraders that consist of a ligand binding to the protein of interest (POI), a second ligand specifically interacting with a Ub ligase, and a linker connecting both ligands (3, 60). PROTACs can bind simultaneously to the POI and Ub ligase, leading to proximity of the POI to the Ub ligase, and POI’s ubiquitylation and degradation *via* the UPS. Afterward, PROTACs can recycle themselves to bind another POI and Ub ligase, exhibiting catalyst-like properties.

Since there are many excellent reviews related to PROTACs already, we would not get into PROTACs in great detail. Instead, we want to emphasize a few PROTAC-related new technologies. Cheng *et al.* (61) introduced the hypoxia-activated leaving group into the structure of an EGFR^Del19^-based PROTAC to develop a kind of hypoxia-activated proteolysis targeting chimera. Using this strategy, they demonstrated a significantly enhanced degradation activity against EGFR^Del19^ in hypoxic conditions compared with normoxic conditions in HCC4006 cells. Light was also employed in the creation of opto-PROTAC (62, 63, 64). To control PROTAC activity by light, Naro *et al.* (62) placed photolabile protecting groups into PROTAC to create opto-PROTAC. Based on this strategy, opto-dBET1 and opto-dALK were synthesized to degrade BET1 and anaplastic lymphoma kinase (ALK) under a light-inducible manner, respectively. Similarly, Liu *et al.* (63) and Reynders *et al.* (64) added photoswitch into PROTACs to develop photochemically targeting chimeras (63, 64). By coupling the photoswitch, photochemically targeting chimeras exhibit little activity and are less toxic in the dark but are activated by the blue-violet light or pulse irradiation leading to the degradation of targeted proteins. To avoid potential toxicity of PROTACs in normal cells, it is important to control on-target degradation of PROTACs precisely in disease cells. Cancer cells often express specific receptor proteins on their cell surface. Antibodies of cell surface receptors have been considered as great weapons for cancer therapy. These antibodies could be conjugated to PROTACs to create cancer-specific PROTACs to kill cancer cells specifically using an “one stone, two birds” strategy. Using this strategy, Maneiro *et al.* (65) created a trastuzumab-PROTAC and killed HER2-positive breast cancer cells by targeting both Her2 and BRD4. Folate receptor α (FOLR1) is overexpressed in tumor cells of many cancer types, whereas normal tissues or cells have very little or no expression of FOLR1 (66). It is one of the most commonly employed targets for drug delivery into cancer cells (66). Liu *et al.* (67) created a new method called folate-caged PROTAC (67). They ligated folate to three PROTAC molecules (ARV-771, MS432, and MS99) to degrade BRDs, MEKs, and ALK in cancer cells, respectively. At the same time, Chen *et al.* (68) employed the same strategy to construct a folate-caged pomalidomide-based ALK PROTAC, called FA-S2-MS4048, which specifically and effectively degrade ALK fusion proteins in cancer cells. These alternative approaches of PROTACs have expanded the applications of PROTACs and could potentially solve their safety issues.

Although rapid progress has been made in PROTAC design and dozens of PROTAC drugs are in clinical trials, PROTACs usually possess big molecular weight; therefore, their pharmacokinetics (PK) could be a big problem. Because of PROTACs’ catalytic nature, traditional approaches might not be appropriate to accurately evaluate PK and pharmacodynamics of PROTACs. Thus, new methods are urgently needed to evaluate PK and pharmacodynamics of PROTACs.

**Figure 5:**
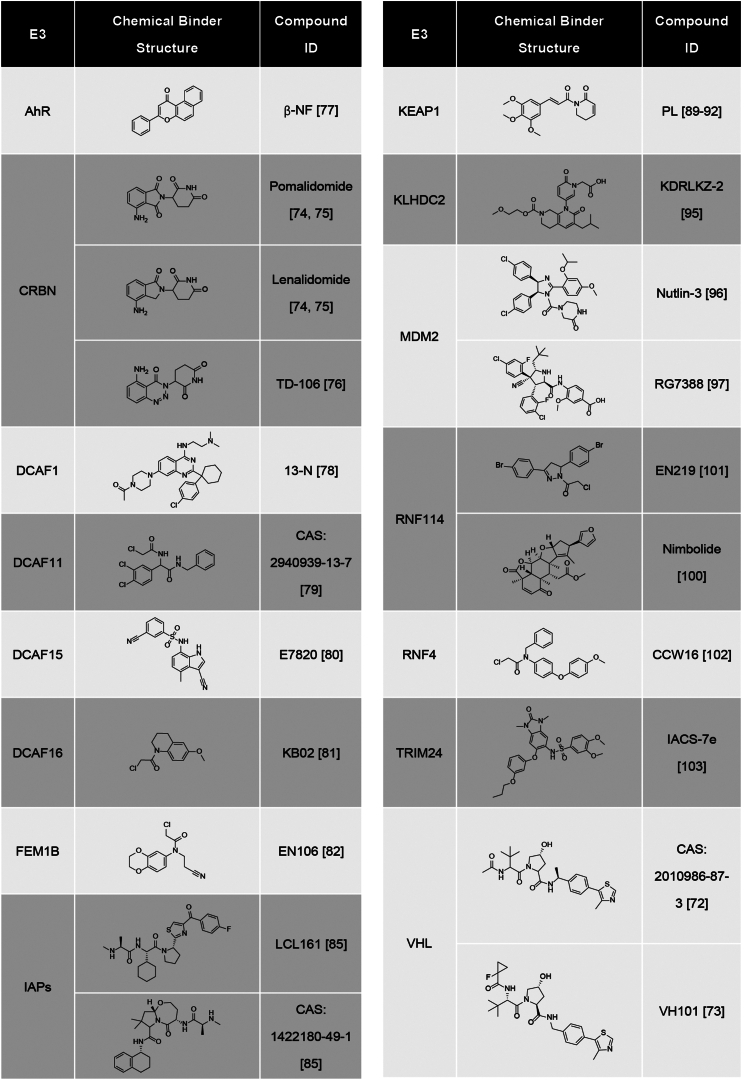
**Ubiquitin ligases and their ligands employed in PROTAC design.** Ubiquitin ligases and structures of their corresponding small-molecule ligands, which have been attempted in construction of PROTACs. PROTAC, proteolysis-targeting chimera.

Another issue is that limited effective ligands of Ub ligases are available for PROTAC design. Small molecule ligand of Von Hippel–Lindau tumor suppressor was among the first, which was employed in design of PROTACs (69, 70, 71, 72, 73). However, molecular glues of CRBN are the most popular ligands in the construction of many PROTACs with excellent degradation capability for diverse substrates (74, 75, 76), because of their small molecular weight and relative safety. In addition, binders of at least 11 other Ub ligases, including AhR, DCAF1, DCAF11, DCAF15, DCAF16, FEM1B, IAPs, Keap1, KLHDC2, MDM2, RNF114, RNF4, and TRIM24, have been attempted in the PROTAC development (77, 78, 79, 80, 81, 82, 83, 84, 85, 86, 87, 88, 89, 90, 91, 92, 93, 94, 95, 96, 97, 98, 99, 100, 101, 102, 103) (Fig. 5). Their clinical applications are needed to be further evaluated. Some of them might be more suitable for development of molecule glue degraders. Further studies are needed to develop powerful E3 ligands from Ub ligases other than CRBN, perhaps from those disease-specific or overexpressed Ub ligases, so their ligands would be disease specific with higher safety.

## Deubiquitylation and DUBTACs

**Figure 6:**
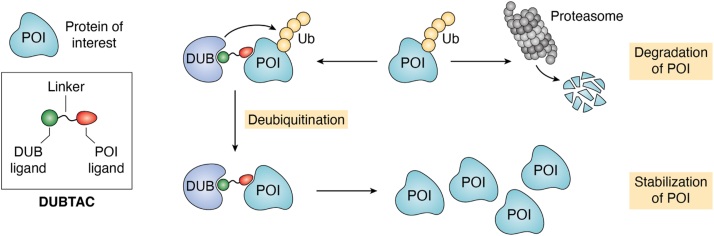
**Mechanism of DUBTACs.** DUBTACs are composed of a deubiquitinase recruiter, a POI ligand, and a linker that connects these two components. By binding POI to the deubiquitinase, DUBTACs can promote the deubiquitylation of the POI and result in the increase of POI protein level. DUBTAC, deubiquitinase-targeting chimera; POI, protein of interest.

Protein ubiquitylation is a reversible process, and conjugated Ub can be removed by a family of deubiquitinases (DUBs) (Fig. 1) (104). The human genome encodes ∼100 DUBs, which play diverse roles in human life and health. PROTACs often target disease-prone proteins that are often overexpressed in malignant cells. However, in certain categories of illnesses, abnormal protein degradation serves as a pathogenic mechanism such as cystic fibrosis transmembrane conductance regulator (CFTR) in cystic fibrosis (105), p53 in cancer cells (106), and insufficient expression of other disease prevention proteins because of accelerated protein degradation (107, 108, 109). In these cases, it would be a therapeutical advantage to focus on stabilizing targeted proteins instead of promoting their degradation. With this goal, Henning *et al.* (7) created a new strategy called DUBTACs, which stabilize unstable proteins by cleaving proteolysis-prone poly-Ub chains on the targeted proteins (Fig. 6). Similar to PROTACs, DUBTACs are heterobifunctional small molecules that consist of a DUB recruiter, a POI ligand, and a linker to connect both parts (7). Rather than degradation, DUBTACs can enhance protein level of POIs by dragging DUB close to the POIs to trigger deubiquitylation (also called deubiquitination) of the POIs.

To enable the DUBTAC platform, Henning *et al.* (7) first identified EN523 as a covalent small-molecule ligand of OTUB1, one member of the OTU family of DUBs, after screening 702 electrophilic small molecules. EN523 can selectively attack the Cys-23 residue of OTUB1, an allosteric cysteine residue, instead of the catalytic residue Cys91, and therefore does not interfere with OTUB1 activity. The authors then created a DUBTAC compound called NJH-2-057, by linking EN523 to lumacaftor through a C5 alkyl linker. Lumacaftor is a small molecule drug to treat cystic fibrosis by reducing misfolding of the ΔF508-CFTR mutant. They found that NJH-2-057 significantly increased the ΔF508-CFTR protein levels by decreasing its ubiquitylation. Using a similar strategy, they found that two DUBTACs, LEB-03-144 and LEB-03-146, with the C3 alkyl linker and the PEG2 linker, respectively, stabilized WEE1 proteins significantly in Hep3B cells. Based on EN523, Liu *et al.* (110) developed a TF-DUBTAC platform aiming at stabilizing tumor-suppressive TFs. In this technology, EN523 was connected to double-stranded DNAs containing consensus DNA sequences for any of three TFs, namely FOXO3A, IRF3, and p53. These three TF-DUBTACs, FOXO-DUBTAC, IRF-DUBTAC, and p53-DUBTAC, were found to stabilize FOXO3A, IRF3, and p53 in tumor cells, respectively, in an OTUB1-dependent manner. These results suggested that targeted protein stabilization could be a promising strategy for cancer therapy.

Although DUB ligands should not directly inhibit the enzymatic activities of DUBs, they would alter the substrate specificity of DUBs, because DUB ligands would prevent the entry of original substrates of DUBs. Thus, the safety of DUBTACs should be paid attention to. Moreover, it is inevitable that DUBTACs could produce drug resistance at the point of DUBs, especially when applied to cancer patients. Considering that the human genome encodes ∼100 DUBs (104), it is necessary to develop more ligands from different DUBs to overcome drug resistance of DUBTACs. Thus far, only a single ligand from one DUB, that is, OTUB1, has been employed in DUBTAC design. In addition to OTUB1, other DUBs could specifically cleave degradation-prone poly-Ub chains as well, such as cezannes and members of the Mindy family DUBs specifically for the K11 and the K48 poly-Ub chains, respectively (111, 112, 113); as well as A20 and VCPIP for both the K11 and the K48 poly-Ub chains (114). These DUBs would be ideal candidates to develop more DUB ligands for the application of DUBTACs.

DUB ligands could also be employed to design TPD drugs. The stability of certain proteins is increased by the K63 poly-Ub chain, which is a nonproteolytic poly-Ub chain (115, 116, 117). One possibility is to employ ligands of certain DUBs, which specifically cleave the K63 chain, to design TPD molecules. These TPD molecules could induce degradation of disease-promoting proteins, such as oncoproteins. For example, Snail and Slug have been implicated in malignancy of cancers. The Pelino-1 Ub ligase conjugates the K63 poly-Ub chains on Snail and Slug, thereby prolonging their half-life (117). One potential strategy to control the oncogenic activities of Snail and Slug is to destabilize them using the K63-specific DUBTAC strategy. Several DUBs digest the K63 poly-Ub chain specifically, such as AMSH, BRCC36, and OTUD1 (114, 118, 119), although there is no ligand available to design the K63 DUBTACs.

The DUBTAC technology is still in its early stage of development. Thus far, only a single ligand of one DUB has been attempted in the DUBTAC design. Moreover, it is still not clear whether the EN523-based DUBTACs could be developed into safe drugs. Other than K11, K48- and K63-specific DUBs, ligands of those M1, K6, K27, K29, or K33-specific DUBs could also be considered for DUBTAC applications.

## Discussions and perspectives

**Figure 7:**
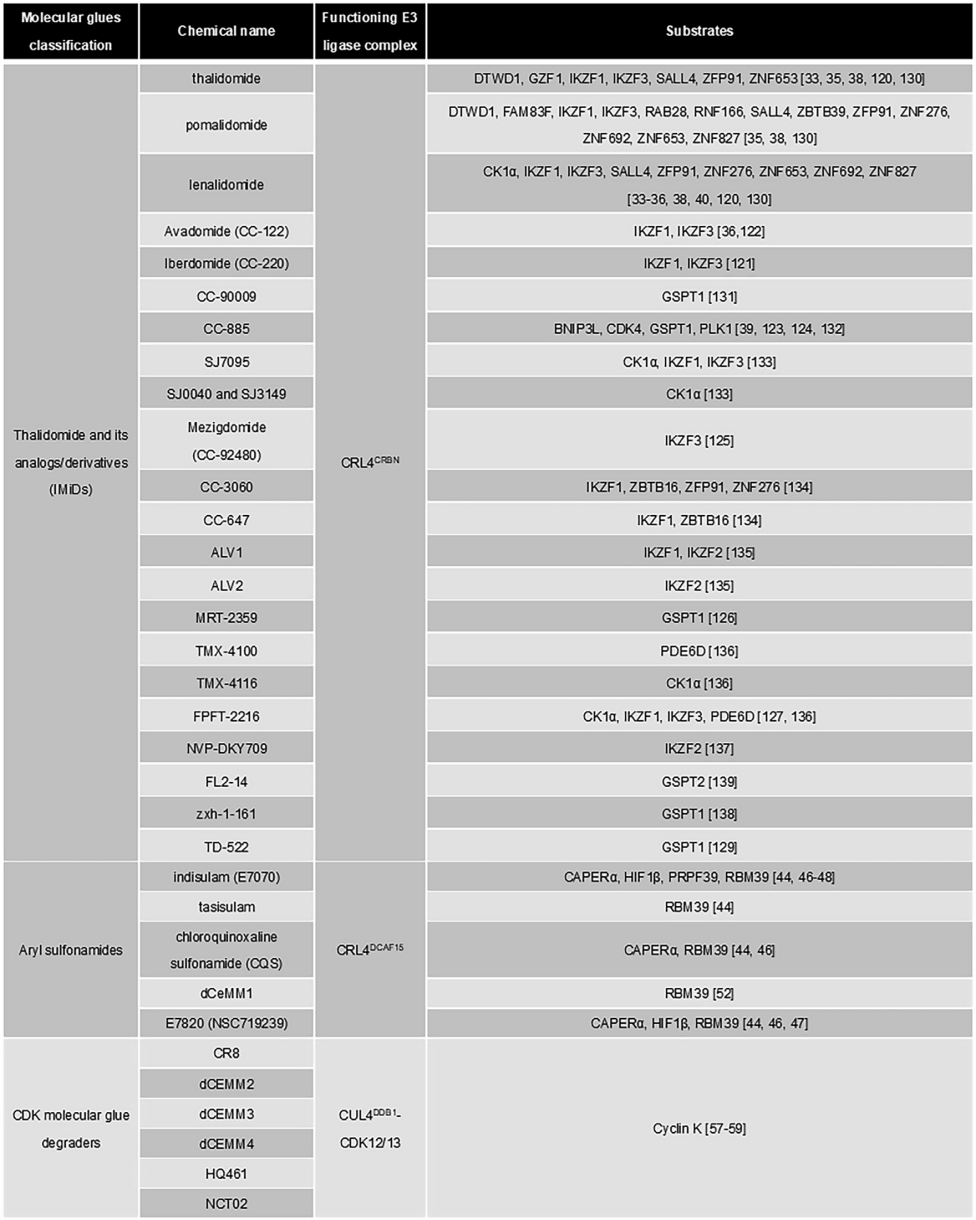
**Molecular glues and their substrates.** Proteins whose degradation was validated are listed in the figure.

Molecular glue degraders and PROTACs are monovalent or heterobifunctional small-molecule CIPs, respectively. Both of them chemically induce the proximity of targeted proteins with Ub ligases to promote ubiquitylation and then degradation of specific proteins, especially disease-relevant ones, *via* the UPS. Both of them possess some advantages and disadvantages. Molecular glue degraders usually possess good pharmacological properties but are hard to be rationally designed. Thus far, the most effective way in discovery of new molecular glue degraders and novel targets is to modify chemical structures of those good degraders and to enhance degradation efficiency of old targets (120, 121, 122, 123, 124, 125, 126, 127, 128, 129) or to identify new targeted proteins using a quantitative MS approach (130, 131, 132, 133, 134, 135, 136, 137, 138, 139, 140, 141) (Fig. 7). Indeed, many good molecular glue degraders are analogs and derivatives of IMiDs and other molecular glue degraders. Conversely, PROTACs can be rationally designed but are usually very large, making their PK a major obstacle as ideal drugs. Due to their high molecular weights and complicated chemical structures, PROTACs could have high tendency to produce off-target issues. As TPD drugs, PROTACs and molecular glue degraders could overcome drug-resistant problems of targeted proteins, since targeted proteins get degraded and face no selection pressure to gain resistant mutations. However, they still encounter resistant issues because of mutations in Ub ligases or closely related catalytic proteins (142, 143). Recent study also indicated that USP15 is a key DUB causing IMiD resistance since USP15-overexpressed cancer cells are often insensitive to IMiD-induced protein degradation (144). Therefore, it is necessary to develop new ligands for Ub ligases other than CRBN and Von Hippel-Lindau Tumor Suppressor for designing PROTACs. In the case of molecular glue degraders, they should possess better drug properties in comparison with PROTACs, but it is still difficult to do rational design of molecular glue degraders on specific targets. Nevertheless, TPD drugs, including PROTACs and molecular glue degraders, are the hope to develop drugs targeting those undruggable disease-prone proteins.

On the contrary, DUBTACs employ heterobifunctional small-molecule CIPs to bring targeted proteins to the proximity of DUBs, deubiquitylate protein substrates, and then enhance their half-lives. So far, EN523 is the only ligand of OTUB1 available. One question to be answered is whether EN523 could function as a molecular glue to stabilize neosubstrates of OTUB1 and whether its neosubstrates belong to disease-relevant proteins or important proteins that cannot be touched. New ligands of OTUB1 or different DUBs are needed to promote the development of DUBTACs. Keeping this in mind, DUBs can not only cleave proteolysis-prone poly-Ub chains to stabilize protein substrates but also chop off nonproteolytic poly-Ub chains from certain substrates. Therefore, ligands from those DUBs that specifically catalyze nonproteolytic poly-Ub chains could either trigger targeted protein turnover or inhibit certain signaling pathways, which rely on nonproteolytic poly-Ub chains for biological activities. DUBTAC is still under early development, but it should provide hope for therapy of certain diseases.

## Conflict of interest

The authors declare that they have no conflicts of interest with the contents of this article.
